# Mind the gap: gender differences in Attention Deficit and Hyperactivity Disorder

**DOI:** 10.1192/j.eurpsy.2025.576

**Published:** 2025-08-26

**Authors:** F. Ramalheira, F. Gonçalves, S. Vieira, R. Cohen, M. Cameira, P. Robalo

**Affiliations:** 1 Hospital Júlio de Matos, ULS São José, Lisbon, Portugal

## Abstract

**Introduction:**

Attention Deficit and Hyperactivity Disorder (ADHD) affects both males and females, however, sex differences can be found in presentation, epidemiology and even influence clinical management. Male-to-female ratio is different in childhood from adulthood, meaning girls with ADHD are probably less referred to medical care and underdiagnosed. Women with ADHD have more prevalence of depression and anxiety than men. Also, fluctuating levels of estrogen and progesterone interferes in symptoms and medication response

**Objectives:**

To study sex differences regarding sociodemographic, mental health care access, and psychiatric comorbidity in a sample of patients from our ADHD outpatient clinic

**Methods:**

We collected data from all patients who attended the Adult ADHD Outpatient Clinic of our hospital from 2017-2022 (N = 262), excluding those without written information or an ICD-11 diagnosis of 6A05 - attention deficit hyperactivity disorder (n=209). We performed a descriptive statistical analysis comparing male (n=132) and female (n=76) on sociodemographic factors, educational achievement, age of diagnosis, treatment and comorbidities

**Results:**

Average of age was 39,4 for females (F) and 34,3 for males(M). Levels of primary education were 5% for both, secondary education 41% F and 53% M, and tertiary education 41% F vs 37% M. 30% F and 37% M had failed at least once during their academic path. 26% F vs 25% M were students, 45% F vs 48% M were working actively and 8% F vs 15% M were unemployed. Only 8% F had an ADHD diagnosis during childhood and adolescence whether 41% of M had a history of early diagnosis and/or treatment. At least once psychiatric comorbidity was found in 75% F and 67% M, and medical comorbidities were present in 36%F and 44% M. Comorbid psychiatric diagnosis were anxiety disorders (36% F vs 26% M), depressive disorders (29% F vs 18% M), intellectual developmental disorders (5% F vs 13% M), substance abuse disorders (5% F vs 9% M), bipolar disorder (11% F vs 5% M), and autism spectrum disorders (3% F vs 5% M). In F, 75% were treated with stimulants and 11% with non-stimulants as in M 80% were treated with stimulants and 8% with non-stimulants. 37% F vs 24% M maintain follow-up, while 50% F vs 61% M abandoned it

**Conclusions:**

In our study, women were less diagnosed in childhood and adolescence than men, regardless of failing in school in a similar percentage, which reflects underdiagnose in girls. Women had more percentage of psychiatric comorbidities, including anxiety, depressive, and bipolar disorders, whereas men had more prevalence of substance abuse and intellectual developmental disorders, meaning that women with ADHD are more prone to develop mood-related comorbidities than men. The percentage of follow-up abandon is also lower on women which indicates that, in spite of being less referred to medical care for ADHD, they are probably more likely to adhere to treatment

**Disclosure of Interest:**

None Declared

